# Monoclinic distortion, polarization rotation and piezoelectricity in the ferroelectric Na_0.5_Bi_0.5_TiO_3_

**DOI:** 10.1107/S2052252518006784

**Published:** 2018-06-01

**Authors:** Hyeokmin Choe, Johannes Bieker, Nan Zhang, Anthony Michael Glazer, Pam A. Thomas, Semën Gorfman

**Affiliations:** a Department of Physics, University of Siegen, Walter-Flex Strasse 3, Siegen 57072, Germany; b Institute of Electromechanical Design, Technische Universität Darmstadt, Darmstadt, Germany; c Electronic Materials Research Laboratory, Key Laboratory of the Ministry of Education and International Center for Dielectric Research, Xian Jaotong University, Xian, People’s Republic of China; d Physics Department, University of Oxford, Clarendon Laboratory, Parks Road, Oxford OX1 3PU, England; e Department of Physics, University of Warwick, Gibbet Hill Road, Coventry CV4 7AL, England; f Materials Science and Engineering, Tel Aviv University, Wolfson Building for Mechanical Engineering, Tel Aviv 6997801, Israel

**Keywords:** ferroelectrics, piezoelectrics, time-resolved X-ray diffraction, polarization rotation

## Abstract

The relationship between crystal structure and physical properties in the ferroelectric Na_0.5_Bi_0.5_TiO_3_ (NBT) has been of interest for the last two decades. Originally, the average structure was held to be of rhombohedral (*R*3*c*) symmetry with a fixed polarization direction. This has undergone a series of revisions, however, based on high-resolution X-ray diffraction, total neutron scattering, and optical and electron microscopy. The recent experimental findings suggest that the true average symmetry is monoclinic (space group *Cc*), which allows for a rotatable spontaneous polarization. Neither polarization rotation nor its potentially important real role in enhanced piezoelectricity is well understood. The present work describes an *in situ* investigation of the average monoclinic distortion in NBT by time-resolved single-crystal X-ray diffraction under external electric fields. The study presents a high-resolution inspection of the characteristic diffraction features of the monoclinic distortion – splitting of specific Bragg reflections – and their changes under a cyclic electric field. The results favour a model in which there is direct coupling between the shear monoclinic strain and the polarization rotation. This suggests that the angle of polarization rotation under a sub-coercive electric field could be 30° or more.

## Abstract 2

Time-resolved high-resolution X-ray diffraction has been implemented to investigate how monoclinic distortion in the ferroelectric Na_0.5_Bi_0.5_TiO_3_ responds to an external electric field. The results strongly favour the model of electric-field-induced polarization rotation and predict that even a sub-coercive electric field can change the direction of the spontaneous polarization vector by 30° or more.

## 1. Introduction   

Na_0.5_Bi_0.5_TiO_3_ (NBT), a perovskite-based ferroelectric, has been the focus of attention for over two decades (Vakhrushev *et al.*, 1985 ▸; Roleder *et al.*, 2002 ▸; McQuade & Dolgos, 2016 ▸). NBT is of interest because of its potentially important role as an end member of many lead-free substitutes to replace the commercially dominant PbZr_1−*x*_Ti_*x*_O_3_ piezoelectric (Takenaka *et al.*, 1991 ▸, 2008 ▸; Shrout & Zhang, 2007 ▸; Panda, 2009 ▸; Rödel *et al.*, 2009 ▸). NBT is also an interesting model system in the crystallography of distorted perovskites. Phase transitions in NBT are realized through symmetry-lowering shifts of the *A*/*B* cations and tilting of the TiO_6_ octahedra. This symmetry-lowering results in the formation of domains that are spontaneously polarized, electromechanically active and switchable by an external electric field. Although all these phenomena are ubiquitous in many other perovskite-based materials (Mitchell, 2003 ▸), NBT is one of the most complex and a number of unresolved controversies remain. The structure–property relationships of NBT continue to be the subject of debate and prompt continued research work on this unusual material.

The commonly accepted crystallographic reference for NBT comes from the neutron powder diffraction work of Jones & Thomas (2002 ▸). This work reported a transformation from an average rhombohedral (*R*3*c*) to an average tetragonal (*P*4*bm*) phase at ∼580 K, following the reorientation of the spontaneous polarization from the body-diagonal to the cell-edge direction (the pseudocubic cell setting with the lattice parameter *a* ≃ 3.9 Å is used for lattice directions and reflection indices throughout this paper.). Most surprisingly, the TiO_6_ octahedra undergo a change in their tilt system from 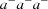 to 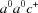 (according to Glazer’s notation system; Glazer, 1972 ▸). The absence of a displacive path for such a phase transition is expressed by the lack of any group–subgroup relationship between the *R*3*c* and *P*4*bm* space groups. In some respects, this thermally driven *R*–*T* phase transformation resembles the compositionally driven one in PbZr_1−*x*_Ti_*x*_O_3_ (PZT) at the morphotropic phase boundary (MPB, *x* ≃ 0.48; Jaffe *et al.*, 1954 ▸, 1971 ▸). In PZT, the Zr-rich side has been recognized as monoclinic rather than rhombohedral close to the MPB, although recent work has shown that the structure is extremely complex, with mixing of disordered and ordered monoclinic regions (Noheda *et al.*, 1999 ▸; Yokota *et al.*, 2009 ▸; Zhang *et al.*, 2011 ▸, 2014 ▸, 2018 ▸; Gorfman *et al.*, 2011 ▸).

Gorfman & Thomas (2010 ▸) have reported a high-resolution X-ray diffraction study of multi-domain NBT ‘single’ crystals. They demonstrated that the angular separation between the different components of the {*hkl*}^1^ Bragg reflections (each component is diffracted from a similarly oriented set of ferroelastic domains) violates rhombohedral *R*3*c* symmetry and favours monoclinic *Cc* average symmetry instead. This average monoclinic symmetry of room-temperature NBT has since been confirmed by many other authors (Aksel *et al.*, 2011 ▸; Ma *et al.*, 2013 ▸; Gorfman *et al.*, 2012 ▸; Kitanaka *et al.*, 2014 ▸; Levin & Reaney, 2012 ▸). The most important implication of monoclinic symmetry is that it allows the polarization to rotate (Vanderbilt & Cohen, 2001 ▸) and thus leads to greater susceptibility to an external electric perturbation (Fu & Cohen, 2000 ▸). The direct effect of the polarization rotation on the lattice parameters might be the reason for the strong electromechanical coupling in both NBT and PZT. For the case of NBT, the true nature of the monoclinic symmetry remains controversial, especially as the average structure is reported to be different from the local one (Aksel *et al.*, 2013 ▸). Neutron scattering studies of pair-distribution function in NBT by Keeble *et al.* (2013 ▸) support the local monoclinic symmetry of the Bi positions, where Bi atoms may displace in two different ‘monoclinic’ directions. At the same time, extended X-ray absorption fine structural studies by Rao *et al.* (2016 ▸) show that the local symmetry of the Bi sites is rhombohedral (consistent with *R*3*c*), while the apparent monoclinic symmetry averages out the combination of different orientation variants of unit cells of rhombohedral symmetry. Regardless of whether the apparent monoclinic distortion has a true local-scale origin or results from the averaging, attempts to test the susceptibility of the monoclinic phase ‘in action’ (dynamically under an electric field) are still rare and mainly limited to the use of static electric fields (Ogino *et al.*, 2014 ▸; Kitanaka *et al.*, 2014 ▸).

The aim of this work is to investigate the apparent monoclinic distortion in NBT under an alternating sub-coercive (<14 kV cm^−1^) external electric field, test the polarization rotation and clarify if this rotation can give rise to high piezoelectricity. We have implemented a stroboscopic data-acquisition system and high-resolution X-ray diffractometer (beamline P08 at the PETRA III storage ring) to collect reciprocal-space maps (RSMs) around the family of most representative Bragg reflections (Gorfman & Thomas, 2010 ▸). We have observed that the monoclinic splitting is indeed strongly sensitive to the external electric field: electric-field-induced shifts of the peaks amount to a piezoelectric effect of as much as 124 pC N^−1^. The positions of the Bragg peaks in reciprocal space are consistent with the existence of 12 monoclinic domains, in which the polarization vector rotates in one of the 12 monoclinic {110} mirror planes. Most importantly, we report that the average shear lattice strain is nonlinear with electric field and this nonlinearity can be well accounted for by the polarization rotation, with the maximum angle of polarization rotation reaching 35°.

## 2. Experimental details   

The NBT single crystal was grown at the Shanghai Institute of Ceramics by the top-seeded solution-growth method (as described by Ge *et al.*, 2008 ▸) and doped with Mn. The crystal was cut to a 0.5 mm thick plate with the surface parallel to (001) and the edges along the [110] and [100] crystallographic directions. Thin (∼100 nm) gold electrodes were sputtered onto the faces to apply the electric field along [001]. We designed a sample stage, which serially connects the electrodes with a high-voltage supply *via* a 1 kΩ active-probe monitor of the capacitive current. The current and polarization hysteresis loops were monitored continuously during the measurement.

**Figure 1:**
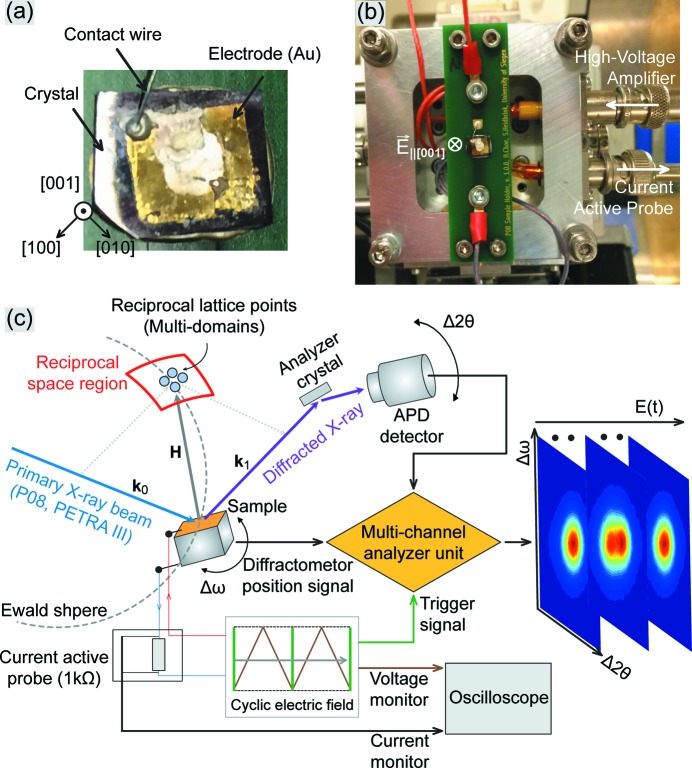
The experimental setup for stroboscopic high-resolution reciprocal space mapping. (*a*) As-grown and prepared sample, with the crystallographic orientation of the edges shown. (*b*) A view of the sample holder for application of the electric field. (*c*) A flow chart of the stroboscopic data-acquisition process. The diffracted signal is collected using a single-photon-counting APD detector placed behind an Si(111) analyser crystal in ω *versus* 2θ scan mode. A cyclic 100 Hz triangular-shaped electric field is applied to the sample. Detector signals are processed using a custom-built stroboscopic data-acquisition system.

Fig. 1 ▸ shows the experimental equipment on the P08 high-resolution four-circle diffractometer at the PETRA III storage ring. The arbitrary function generator (HMF-2550, Hameg) and high-voltage amplifier (AMT-3B20, Matsusada) produce a triangular-shaped AC high-voltage signal/electric field with an amplitude of 14 kV cm^−1^. This field is significantly smaller than the coercive field of ∼45 kV cm^−1^ reported for an Mn-doped NBT single crystal (Ge *et al.*, 2010 ▸). We used an avalanche photodiode (APD) single-photon counting detector and Si(111) analyser crystal and measured the scattering intensity as a function of ω (rocking) and 2θ (scattering) angles around the [004]* position of reciprocal space. The output of the APD detector was introduced directly into a custom-built stroboscopic data-acquisition system (Gorfman *et al.*, 2010 ▸, 2013 ▸; Gorfman, 2014 ▸; Choe *et al.*, 2015 ▸, 2017 ▸). The system implements the working principle of a multi-channel analyser: it assigns detector counts to one of 10 000 time channels, where each channel has a fixed time delay to the beginning of latest high-voltage cycle. Each point in the RSM was collected for 10 s = 1000 electric-field cycles. The frequency of the applied electric field was 100 Hz and the time resolution (channel width) was 1 µs. The X-ray energy was set to 15.1 keV (λ = 0.827 Å), just below the ‘Bi’ *L*_2_ absorption edge, giving an average penetration depth for the measured reflection of 〈*t*〉 = sinθ/2μ = 5.3 µm. This is ∼2.5 times deeper than the penetration depth which was previously used in the experiment of Gorfman & Thomas (2010 ▸).

## 3. Results   

**Figure 2:**
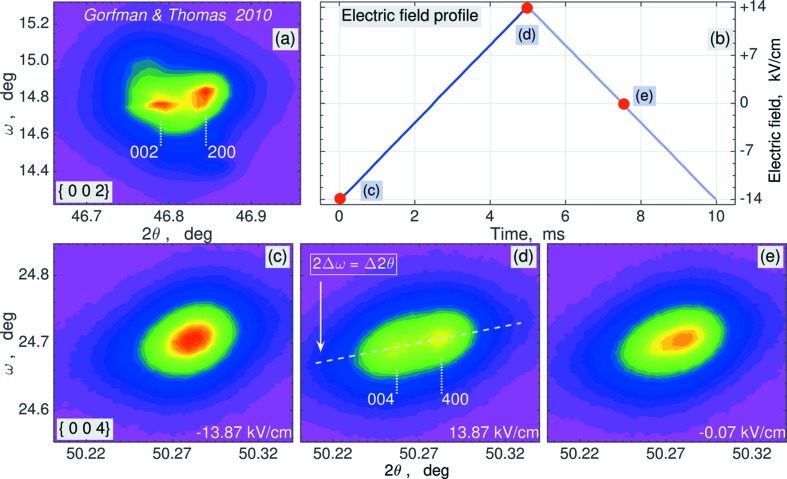
The intensity of the X-ray scattering around the {00*h*} family of reflections. (*a*) Static ω *versus* 2θ mesh of the {002} reflections family, regenerated from the earlier data set of Gorfman & Thomas (2010 ▸). (*b*) The time-dependence of the applied external electric field. (*c*)–(*e*) Stroboscopically collected ω *versus* 2θ meshes of the {004} family of reflections, corresponding to different time channels and electric fields. The splitting along the 2θ axis violates the trigonal symmetry of the pseudocubic lattice. This splitting is commonly recognized as a ‘fingerprint’ of the *M_A_*/*M_B_* monoclinic distortion in perovskites (see *e.g.* Noheda *et al.*, 1999 ▸). An animated version of panels (*c*)–(*e*) is available in the supporting information.

Fig. 2 ▸(*a*) reproduces an ω *versus* 2θ RSM of one of the {002} reflections from our previous studies (Gorfman & Thomas, 2010 ▸) (measured using a home-laboratory high-resolution PANalytical MRD diffractometer). This RSM contains two Bragg peaks, separated along the 2θ axis and diffracted from two families of ferroelastic domains. Different 2θ angles mean different lengths of the corresponding reciprocal-lattice vectors [*H* = (2sinθ)/λ]. Accordingly, such splitting violates the rhombohedral symmetry of the domains, which would constrain the average pseudocubic lattice parameters to be *a* = *b* = *c* and α = β = γ. The work of Gorfman & Thomas (2010 ▸) reports on the detailed analysis of such splitting in many other families of reflections, which includes 41 different RSMs. The results of this work clearly suggested that the above constraint must be lifted to *a* = *b* 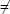 *c* and α = β 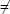 γ, corresponding to an average monoclinic lattice with a mirror plane || to 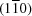 2. The structure of room-temperature NBT must therefore be described by the monoclinic *Cc* space group, which is a subgroup of rhombohedral *R*3*c*.

**Figure 3:**
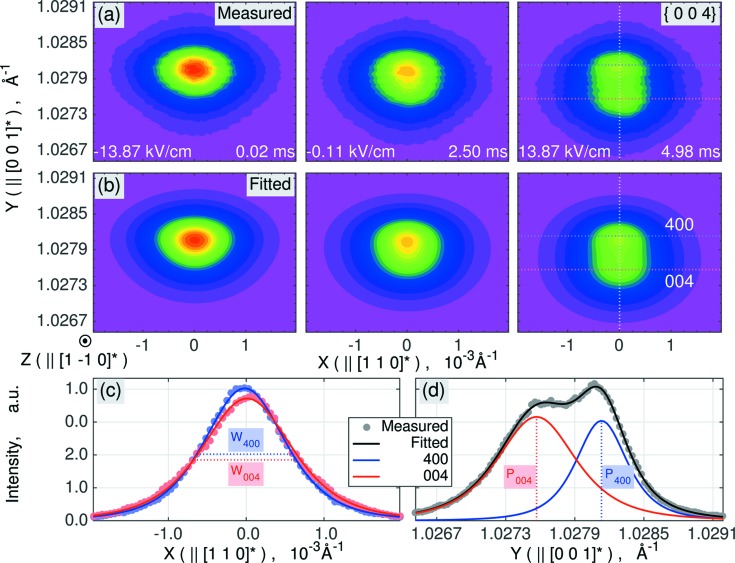
Measured and fitted X-ray scattering intensity distributions around {004} reflections in the reciprocal lattice coordinates *X* || [110]* and *Y* || [001]*. (*a*) Measured RSMs and (*b*) their fit by the superposition of a pair of two-dimensional Moffat distribution functions. (*c*) and (*d*) *X* and *Y* intensity profiles of 400 (higher) and 004 (lower) Bragg-peak components at 13.87 kV cm^−1^ of electric field. An animated version of panels (*a*) and (*b*) is available in the supporting information.

In the present work, we selected the most representative set of Bragg reflections to measure the field dependence of the monoclinic distortion. Figs. 2 ▸(*c*)–2 ▸(*e*) display stroboscopically collected RSMs of the {004} reflection (measured on the P08 beamline at PETRA III), corresponding to three different time channels or electric field states, marked by the circles in Fig. 2 ▸(*b*). Each RSM contained 5928 intensity values on the mesh of 78 × 76 points along the ω and 2θ directions, respectively. An animated set of 250 RSMs (after binning of every 40 channels to improve the counting statistics) is available in the supporting information. The varying separation of peaks on the 2θ axis suggests that the monoclinic distortion is field dependent. Fig. 3 ▸(*a*) shows three RSMs of an {004} reflection in Cartesian coordinates, with the horizontal axis *X* || [110]* and the vertical axis *Y* || [001]*, where *X* and *Y* are the coordinates of the scattering vector. Both the *X* and *Y* axes on these maps lie in the diffraction plane and the *Y* axis is parallel to the scattering vector. This *Y* axis corresponds to the Δ(2θ) = 2(Δω) dashed line in Fig. 2 ▸(*d*). The open slit of the detector is perpendicular to the diffraction plane, thus giving rise to automatic intensity integration along the *Z* 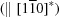 direction.

We have fitted these RSMs using the superposition of two Moffat two-dimensional distribution functions, each of which has adjustable parameters: the positions of the peaks (*x*_0_, *y*_0_), full widths at half maxima (σ_*x*_, σ_*y*_), integrated intensities *I* and shape parameter β. This means we used 12 model parameters to describe the intensity distribution over 5928 points on each RSM. The details are given in the supporting information. Fig. 3 ▸(*b*) shows three RSMs, calculated using the best-fit values of the parameters. The graphs in Figs. 3 ▸(*c*) and 3 ▸(*d*) then cut reciprocal space along the *X* and *Y* directions, clearly showing the *Y* separation of the peak components. An animated version of this figure (in the supporting information) shows that the split peaks have significantly different time and electric-field dependencies.

**Figure 4:**
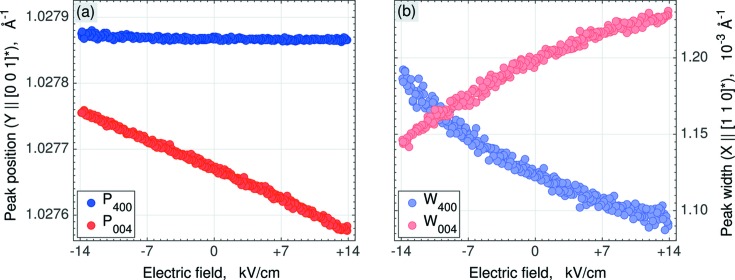
Best-fit values of the ‘position’ and ‘width’ parameters of the 004 (red) and 400 (blue) reflections as a function of time and applied electric field. (*a*) The *Y* component of the Bragg-peak positions *P*_004_ and *P*_400_. (*b*) The *X* component of the Bragg-peak widths *W*_004_ and *W*_400_.

Finally, Fig. 4 ▸ shows the field dependence of the key model parameters for both contributing Bragg peaks. These key parameters are the peak positions (Figs. 4*a* and 4*b*) and peak widths (Figs. 4*c* and 4*d*) along *Y* and *X*, respectively. In the following we will introduce this model, which will help us to calculate the monoclinic distortion parameters as a function of electric field.

## 4. Modelling of the field dependence of RSMs   

### 4.1. Monoclinic distortion, polarization rotation and monoclinic domains   

**Figure 5:**
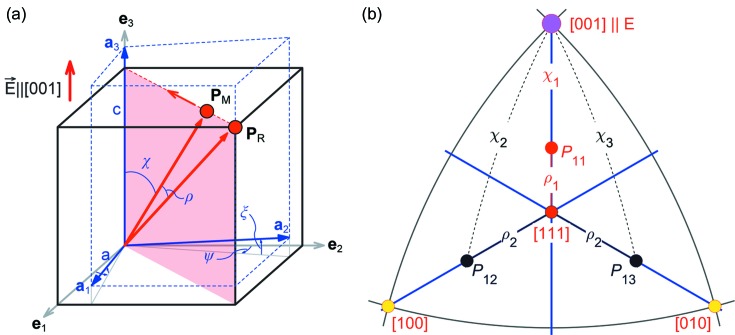
The distortion of the monoclinic unit cell and the polarization rotation. (*a*) A schematic showing the monoclinic distortion of the pseudocubic unit cell due to the rotation of the polarization direction. The angle between the polarization vector and the cell-body diagonal is ρ. The unit cell is modelled using four ρ-dependent parameters (*c*, *a*, ψ and ξ): *c* and *a* are the lengths of the unit-cell edges in and out of the polarization rotation plane, respectively, and ψ and ξ are the unit-cell shear angles. (*b*) A stereographic projection of polarization rotation planes and directions of polarization *P*_11_, *P*_12_ and *P*_13_ in the monoclinic domains *M*_11_, *M*_12_ and *M*_13_, respectively. An animated version of panel (*a*) is available in the supporting information.

We now discuss whether the measured changes in the peak positions and widths may be explained by a model of electric-field-dependent monoclinic distortion and polarization rotation. Fig. 5 ▸ shows how the pseudocubic unit cell is distorted after a transition from a cubic, 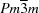 (*a* = *b* = *c* and α = β = γ = 90°), to a monoclinic, *Cc* (*a* = *b* 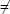 *c* and α = β 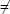 γ), structure. The figure shows the orientation of the pseudocubic basis vectors **a**_1_, **a**_2_, **a**_3_ in a monoclinic domain relative to the Cartesian reference frame **e**_1_, **e**_2_, **e**_3_, aligned with the edges of the cubic unit cell. The monoclinic distortion can be modelled using four free parameters, *c*, *a*, ψ and ξ. Here, *c* and *a* are the unit-cell lengths in and out of the monoclinic mirror plane, respectively, and ψ and ξ are the shearing angles of the unit cell, as shown in Fig. 5 ▸(*a*). We also assume that all these free parameters can be expressed as a function of the polarization rotation angle ρ, the angle between the monoclinic **P**_M_ and rhombohedral **P**_R_ polarization directions. The positive and negative polarization rotation angles correspond to the monoclinic *M_A_* (ρ 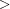 0) and *M_B_* (ρ 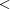 0) phases, respectively (Vanderbilt & Cohen, 2001 ▸; Zhang *et al.*, 2014 ▸). The loss of the three-fold rotational symmetry results in the formation of monoclinic domains, in which the polarization vector rotates in a plane between the unit-cell body-diagonal directions towards one of the three edges (Fig. 5 ▸ *b*). Therefore, a maximum of 24 monoclinic domains can be created in which the polarization rotation angle, ρ, is measured from one of the eight 〈111〉 body-diagonal directions. Because the crystal response is strongly asymmetric with respect to the electric field direction, we must assume that *e.g.* the previous poling history of the sample kept only four rhombohedral domains with the polarization close to the ‘rhombohedral’ [111], 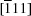, 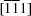 and 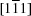 directions, resulting in the formation of only 12 monoclinic domains. We mark these domains as *M_mn_*, where *m* = 1, 2, 3, 4 correspond to **P**_R_ || [111], **P**_R_ || 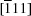, **P**_R_ || 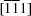 and **P**_R_ || 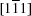, respectively, *n* = 1 corresponds to the domains where the electric field lies in the polarization rotation plane (see Fig. 5 ▸ *b*) and *n* = 2, 3 correspond to the domains where the electric field is directed out of the polarization rotation plane.

### 4.2. Modelling of the positions of the diffraction peaks   

We use the model and definitions above to simulate the positions in the {004} family of reflections, each diffracted from one of the 12 monoclinic domains. To do this we introduce the orientation matrix of a monoclinic domain (Fig. 5 ▸ *a*), 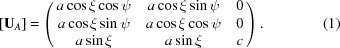
The columns of this matrix are the coordinates of the vectors **a**_*i*_ in the Cartesian coordinate system **e**_*i*_. The reciprocal orientation matrix [**U**_*B*_] (the columns of which are the coordinates of the reciprocal basis vectors 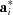, such that 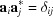) can be obtained as 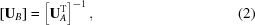
and therefore 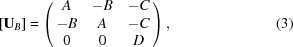
where 
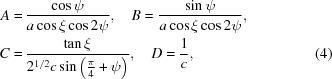
are also the functions of the polarization rotation angle, *A*(ρ), *B*(ρ), *C*(ρ) and *D*(ρ). Transforming the coordinates of the vectors 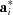 into the laboratory coordinate system *X*, *Y*, *Z* of Fig. 3 ▸ (here *X* || **e**_1_ + **e**_2_, *Y* || **e**_3_ and *Z* || **e**_1_ − **e**_2_) is done using the matrix equation [**U**_*B*_]_*XYZ*_ = [*XYZ*] · [**U**_*B*_] with 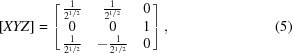
The functional form of the orientation matrices of all other monoclinic domains can be calculated using 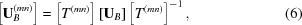
where the columns of the twinning matrices [*T^(mn)^*] (see Table 1 ▸) are the coordinates of the Cartesian cubic axes 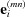 of the domains *M_mn_* in the **e**_*i*_ coordinate system. Finally, the positions of the Bragg peak diffracted from domain *mn* are described by the first two components (*X* and *Y*) of the third column of 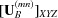. Table 1 ▸ summarizes the twinning matrices for all 12 monoclinic domains and the form of the reciprocal orientation matrices. The arrows indicate the polarization rotation direction induced by an [001]-oriented electric field.

**Figure 6:**
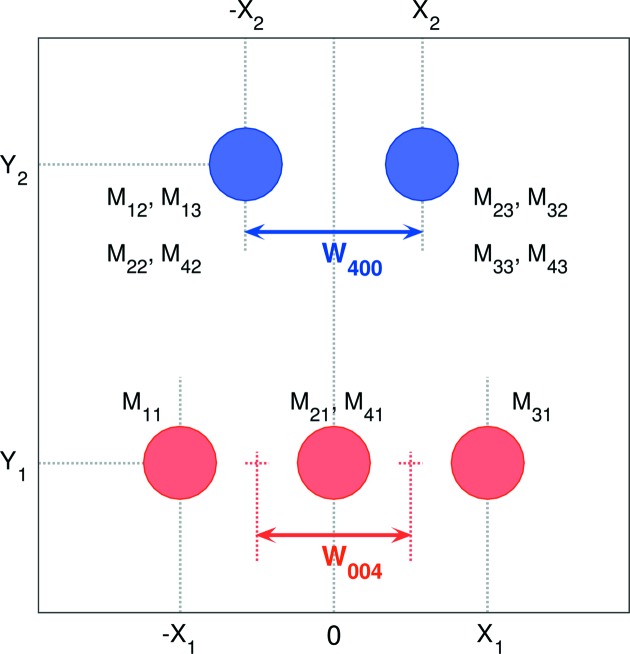
Schematic drawing for the set of {004} reflections, corresponding to 12 monoclinic domains. The positions of all peaks are described by four independent parameters, *X*_1_, *Y*_1_, *X*_2_ and *Y*_2_ (as used in Table 1 ▸), which are directly connected to the monoclinic distortion parameters Δ*c*_1_, Δξ_1_, Δ*a*_2_ and Δψ_2_ by equations (4) in the text. An animated version is available in the supporting information.

Symmetry dictates that the polarization rotation angles are the same for all monoclinic domains from groups *M*_*m*1_ (ρ = ρ_1_) and *M*_*m*2_/*M*_*m*3_ (ρ = ρ_2_). The following notation is introduced in Table 1 ▸: 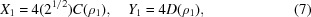


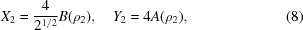
Fig. 6 ▸ represents the right-hand column of Table 1 ▸ in the form of a schematic drawing of the positions of the Bragg reflection, diffracted from all 12 monoclinic domains. Note that the separation of the Bragg peaks along the *X* axis might be significantly smaller than the peak width (arising from *e.g.* crystal mosaicity, later defined as 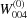 and 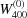), and therefore cannot be seen in Fig. 3 ▸ directly. Instead, this separation can be extracted from the field-dependent peak broadening, displayed in Fig. 4 ▸(*b*). Following equations (7) and (8) and the scheme in Fig. 6 ▸, the broadening can be simulated as
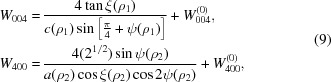
We further assume that the monoclinic distortion angles ψ and ξ are so small that we can replace all the trigonometric functions above by the corresponding first-order Taylor expansions. This brings us to the following expressions, 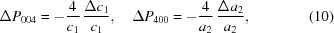


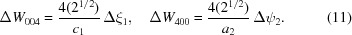
Here, the Δ sign stands for the difference between *e.g.* a zero-state value and a non-zero-field value. The equations allow for the direct evaluation of the monoclinic distortion parameters Δ*c*_1_ = Δ*c*(Δρ_1_) and Δξ_1_ = Δξ(Δρ_1_) (domains *M*_1*n*_), and Δ*a*_2_ = Δ*a*(Δρ_2_) and Δψ_2_ = Δψ(Δρ_2_) (domains *M*_2*n*_ or *M*_3*n*_).

### 4.3. Nonlinear electric field response and the model of polarization rotation   

According to equations (10) and (11), the monoclinic distortion parameters Δξ_1_, Δψ_2_ and Δ*a*_2_ have the same field dependencies as the peak-shape parameters *W*_004_, *W*_400_ and *P*_400_, respectively. Therefore (Fig. 4 ▸), all the derived monoclinic distortions (except for Δ*c*_1_) are essentially nonlinear with respect to the magnitude of the electric field.

**Figure 7:**
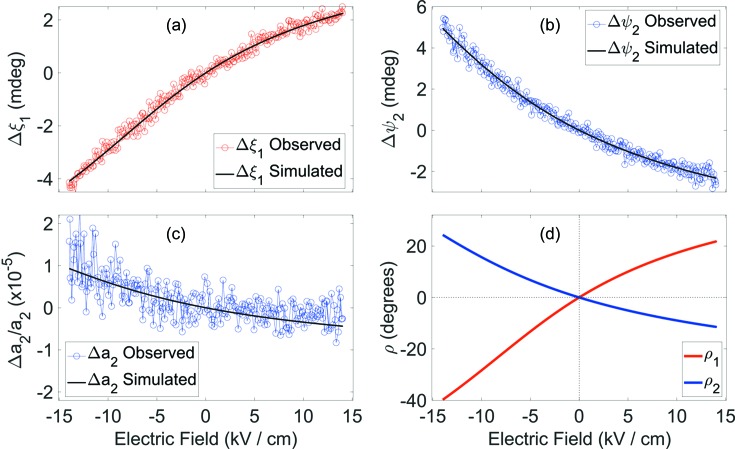
Electric field dependence of the strain parameters and polarization rotation angle. (*a*)–(*c*) Comparison between the observed change in the monoclinic strain parameters Δξ_1_, Δψ_2_ and Δ*a*_2_ (circles) and the calculated change according to equations (15)–(17) [Disp-formula fd17]. (*d*) The polarization rotation angles ρ_1_ and ρ_2_, as defined in Fig. 5 ▸(*b*).

In the following we address the question of whether this nonlinearity can be accounted for by polarization rotation. More specifically, we will test if the monoclinic strains can be described as linear functions of the polarization rotation angle ρ, rather than of the magnitude of the electric field *E*, so that Δξ_1_ = *F*_ξ_Δρ_1_, Δψ_2_ = *F*_ψ_Δρ_2_ and Δ*a*_2_ = *F*_*a*_Δρ_2_. To derive the ρ_1_(*E*) and ρ_2_(*E*) dependence, we shall assume that the free energy Δ*G* (Devonshire, 1954 ▸) has a quadratic dependence on ρ with its minimum at ρ = 0, so that the total free energy (including the term describing the interaction of electric field and spontaneous polarization) is
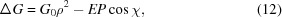
where *G*_0_ is the energy expansion coefficient, *P* is the length of the polarization vector and χ is the angle between the polarization and electric field directions, as marked in Fig. 5 ▸(*b*): 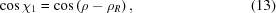


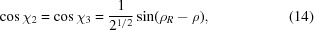
where ρ_*R*_ = arccos(1/3^1/2^) ≃ 54.57° is the angle between the cube edges and the body diagonal. Substituting equations (13) and (14) into (12) and locating the position of the global minimum by equating 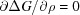 gives the polarization rotation angles Δρ in the domains *M*_*n*1_ and *M*_*n*2_/*M*_*n*3_: 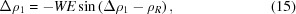


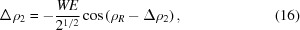
with *W* = *G*_0_/2*P*. Using our assumption that the change in the monoclinic distortion parameters is linear with respect to polarization rotation, we rewrite equations (15) and (16) as 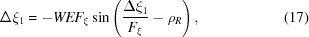


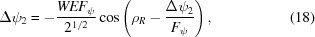


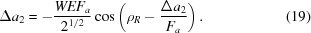
Equations (17)–(19) [Disp-formula fd19] can be solved numerically for any given electric field magnitude *E*, so that the unknown model parameters *W*, *F*_ξ_, *F*_ψ_ and *F_a_* can be found from the best fit to the experimental values. The solutions are shown in Figs. 7 ▸(*a*)–7 ▸(*c*), where both observed [from equations (10) and (11)] and simulated [according to equations (17)–(19) [Disp-formula fd19]] monoclinic distortion parameters Δξ_1_, Δψ_2_ and Δ*a*_2_ are displayed. These figures show that our simplified model above is highly effective in accounting for the nonlinear dependence of all three nonlinear monoclinic distortion parameters. This good match between observed and simulated monoclinic distortion parameters points strongly to a close connection between electric-field-induced polarization rotation and lattice strain, clearly suggesting that the corresponding piezoelectric effects are principally intrinsic rather than extrinsic in origin. Fig. 7 ▸(*d*), however, represents the Δρ_1_ = *F*_ξ_Δξ_1_ and Δρ_2_ = *F*_ψ_Δψ_2_ polarization rotation angles, showing that this nonlinearity must assume rotation of the polarization vector by an angle larger than 35°.

## 5. Low-field piezoelectric coefficients of a single monoclinic domain   

The intrinsic low-field piezoelectric coefficients of a single monoclinic domain can be calculated from the experimental results as *d_ijk_* = 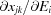 (*E* = 0), where *x_jk_* and *E_i_* are the components of the strain tensor and electric field vector, respectively, in the domain-related Cartesian coordinate system 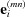. The strain tensor for the monoclinic distortion (Fig. 5 ▸ *a*) is: 
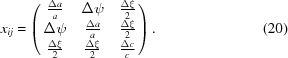
The piezoelectric coefficients *d*_3*jk*_ describe the strain in response to the electric field applied along 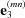 (parallel to the polarization rotation plane). Such orientations are realized in the *M*_*m*1_ domains, so that the field dependence of the monoclinic distortion parameters Δ*c*_1_ and Δξ_1_ can be used to calculate the piezoelectric coefficients *d*_333_ and *d*_323_. Similarly, the first and second rows in the tensor representation of the piezoelectric coefficients *d*_1*jk*_ = *d*_2*jk*_ describe the strain in response to the electric field being parallel to the 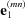 and 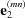 axes (out of the polarization rotation plane). Such orientations of the electric field are realized in the monoclinic domains *M*_*m*2_ and *M*_*m*3_, respectively. Therefore, the field dependence of the monoclinic distortion parameters Δψ_2_ and Δ*a*_2_ gives the piezoelectric coefficients *d*_112_ and *d*_111_, respectively: 
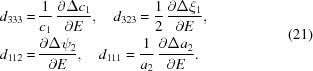
The numerical values of the corresponding piezoelectric constants are *d*_333_ = 124.1 pC N^−1^, *d*_323_ = 20.36 pC N^−1^, *d*_112_ = −43.93 pC N^−1^ and *d*_111_ = 4.29 pC N^−1^. The field dependence of the strain suggests that the low-field piezoelectric coefficients, *d*_323_, *d*_112_ and *d*_111_ are associated with the polarization rotation.

## 6. Discussion   

The macroscopic piezoelectric coefficients of NBT materials (ceramics and single crystals) range between 20 and 100 pC N^−1^ (Ge *et al.*, 2010 ▸; Foronda *et al.*, 2014 ▸; Hiruma *et al.*, 2010 ▸, 2009 ▸). These have the same order of magnitude as the values in the previous paragraph. Therefore, our results suggest that electromechanical coupling in NBT is predominantly intrinsic. The intrinsic character of the electromechanical coupling is seen in the shifts of the angular positions in the {004} family of twin reflections. We have also argued that some components of the strain can be explained straightforwardly by polarization rotation. This suggestion follows from the nonlinear dependence of the monoclinic lattice distortion parameters (Δξ_1_, Δψ_2_ and Δ*a*_2_) on the electric field. We must stress, however, that using a polarization rotation argument to explain this dependence produces an unexpectedly large amplitude for the polarization rotation angle: 25° between [111] and [001] (*M*_*A*_ phase) and up to 35° between [111] and [110] (*M*_*B*_ phase). This would mean that the polarization rotation represents such a ‘soft mode of structural changes’ that even a sub-coercive electric loading of 14 kV cm^−1^ can change it greatly. Can a polarization vector really rotate so much in a ferroelectric crystal? Our preliminary analysis favours a positive answer to this question and might lead to the suggestion of different origins for such extreme ‘structural softness’.

Firstly, the large polarization rotation may originate from the intricate structural flexibility of perovskites, where a ‘soft’ displacement of *A*/*B* cations from the centres of the corresponding oxygen *A*O_12_/*B*O_6_ cages occurs. This explanation assumes that the above displacements are long-range ordered and that the polarization rotates through coherent changes of atomic position in every unit cell of the crystal. Such coherent structural changes would appear as a change in the ‘Bragg intensities’ and could be analysed using the standard structure factor formalism (see *e.g.* Gorfman *et al.*, 2016 ▸, 2013 ▸, 2006 ▸; Tsirelson *et al.*, 2003 ▸; Schmidt *et al.*, 2009 ▸). Indeed, we observe changes in the integrated intensity for both 400 and 004 Bragg peaks by ∼10% (see Fig. S1 in the supporting information) but we have not been able to measure enough Bragg reflection intensities to model the structural changes within the *Cc* space group.

Secondly, the large polarization rotation may originate from variations in the local structure and short-range order parameters. In this model, by contrast with the first, the displacement of the atoms varies from one unit cell to another, so that the polarization can only be defined on average. The strong structural disorder in NBT has been documented by the observation of diffuse X-ray scattering (Kreisel *et al.*, 2003 ▸; Thomas *et al.*, 2010 ▸; Gorfman *et al.*, 2015 ▸) or total neutron scattering (Keeble *et al.*, 2013 ▸). A reverse Monte Carlo simulation of the atomic pair-distribution function in NBT (Keeble *et al.*, 2013 ▸) demonstrated that the Bi atoms in the monoclinic {110} planes do indeed differ and involve two co-existing directions of bismuth displacements. From this starting point, it follows that one might suggest that the application of an external electric field in a particular direction switches a sub-population of atoms, thus changing the distribution of Bi atoms over two metastable states. Such a redistribution would produce a change in the average direction of the polarization vector relatively easily.

Thirdly, the large polarization rotation in NBT is strongly reminiscent of that in the compositionally driven polarization rotation in PbZr_1−*x*_Ti_*x*_O_3_ at the morphotropic phase boundary. The work of Zhang *et al.* (2014 ▸) shows that even a minor change in the composition *x* near the morphotropic phase boundary leads to the rotation of the average direction of the Pb displacement vector by a large angle of well beyond 35°. This large polarization rotation is commonly considered as one of the origins of enhanced piezo-activity at this particular boundary in the phase diagram.

We finally note the ongoing discussion of the true structural origin of the monoclinic phase in NBT. One suggestion is that the monoclinic symmetry can be mimicked by an adaptive phase mechanism (Jin *et al.*, 2003 ▸; Wang, 2007 ▸), which is a microstructural material state made of periodically arranged nano-domains. Here the effective polarization rotation can be driven by the dynamics in the hierarchical nano-domain pattern, where each domain would have rhombohedral symmetry. The polarization direction of such an assembly is given by the volume average of polarization in the individual sets of nano-domains. Provided that the domains are sufficiently small, the Bragg diffraction pattern of such an adaptive structure would be indistinguishable from the equivalent Bragg diffraction pattern from a truly long-range monoclinic phase.

Finally, the demonstrated time-resolved reciprocal-space mapping approach has the potential to uncover the origins of electromechanical coupling in other ferroelectric perovskites.

## Supplementary Material

Animation 1. DOI: 10.1107/S2052252518006784/fc5023sup1.mp4

Animation 2. DOI: 10.1107/S2052252518006784/fc5023sup2.mp4

Animation 3. DOI: 10.1107/S2052252518006784/fc5023sup3.mp4

Animation 4. DOI: 10.1107/S2052252518006784/fc5023sup4.mp4

Additional figure. DOI: 10.1107/S2052252518006784/fc5023sup5.pdf

## Figures and Tables

**Table 1:** Summary of the twinning matrices for all 12 monoclinic domains and the form of the reciprocal orientation matrices Arrows indicate the polarization rotation direction induced by an [001]-oriented electric field.

*D*	Polarization rotation plane and rotation angle	Twinning matrix, *T*	Form of the 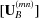 matrix	{004} reflection position *XY*
*M*_11_	[111] → [001], ρ_1_	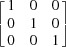	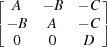	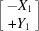
*M*_12_	[111] ← [100], ρ_2_	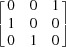	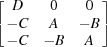	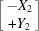
*M*_13_	[111] ← [010], ρ_2_	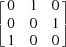	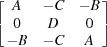	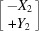
*M*_21_	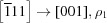	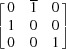	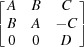	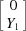
*M*_22_	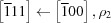	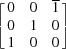	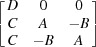	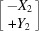
*M*_23_	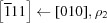	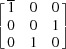	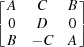	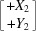
*M*_31_	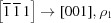	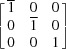	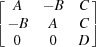	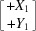
*M*_32_	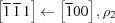	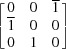	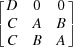	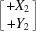
*M*_33_	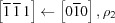	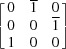	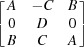	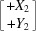
*M*_41_	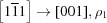	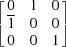	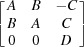	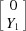
*M*_42_	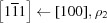	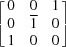	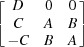	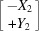
*M*_43_	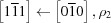	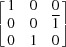	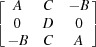	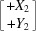

## Footnotes

1 {*hkl*}_PC_ denotes all possible reflections, obtained by applying the cubic *m*3*m* point symmetry group to the reflection *hkl*. For cubic symmetry, the lengths of such reciprocal-space vectors would be the same. However, when the symmetry is distorted the lengths of the vectors vary, giving characteristic splitting of reflections for every crystal system.

2 These lattice parameters will look conventionally monoclinic (*a*_m_ ≠ *b*_m_ ≠ *c*_m_, α_m_ = γ_m_ = 90°, β_m_ ≠ 90°) when the unit-cell vectors are transformed as **a**_m_ = **a** + **b**, **b**_m_ = **a** − **b** and **c**_m_ = **c**. In this case the mirror plane becomes parallel to (010)_m_
