# Supplementary material for: Monoclinic distortion, polarization rotation and piezoelectricity in the ferroelectric Na0.5Bi0.5TiO3
Source: IUCrJ. 2018 Jun 1;5(Pt 4):417–27. doi: 10.1107/S2052252518006784 (PMC6038949; doi:10.1107/S2052252518006784)
Supplement: Supplementary file 5 [file m-05-00417-sup5.pdf]

IUCrJ

Volume 5 (2018)

Supporting information for article:

Monoclinic distortion, polarization rotation and piezoelectricity in the ferroelectric Na0.5Bi0.5TiO3

Hyeokmin Choe, Johannes Bieker, Nan Zhang, Anthony Michael Glazer, Pam A. Thomas and Semën Gorfman

The figure below demonstrates the time dependence of all the model parameters, used for fitting the observed reciprocal space maps by the superposition of two Moffat 2D distribution function:

$$f(x,\,y)\;=\;I\,\frac{4\left(2^{\frac{1}{\beta}}-1\right)(\beta-1)}{\pi\sigma_x\sigma_y}\left[1+\left(4\left(2^{\frac{1}{\beta}}-1\right)\right)\left(\frac{(x-x_0)^2}{\sigma_x^2}+\frac{(y-y_0)^2}{\sigma_y^2}\right)\right]^{-\beta}$$

Here I is the integrated intensity of the peak, x_0 and y_0 are the coordinates of the mass centres of the peaks σ_x and σ_y are their full-widths at half maxima, β is the peak shape parameter.

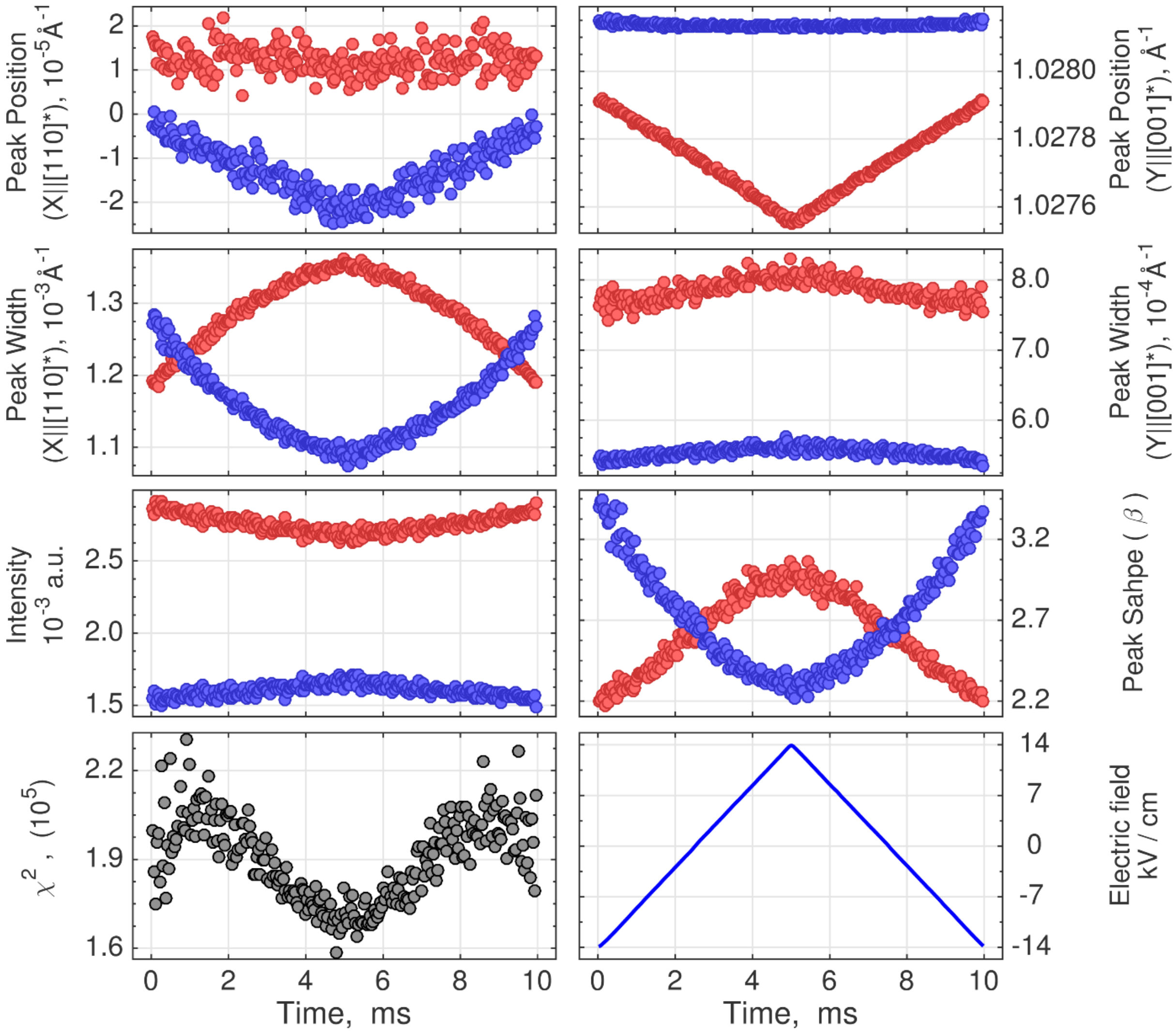

Figure S1 The field and time dependence of all the model parameters for two Bragg peaks in the reciprocal space maps, displayed in Figure 3.
